# Comment on ‘Effect of intraoperative haemoadsorption therapy on cardiac surgery for active infective endocarditis with confirmed *Staphylococcus aureus* bacteraemia’

**DOI:** 10.1093/icvts/ivad051

**Published:** 2023-03-25

**Authors:** Simon A Amacher, Jules Miazza, Martin Siegemund, David Santer

**Affiliations:** Intensive Care Medicine, University Hospital Basel, Basel, Switzerland; Department of Cardiac Surgery, University Hospital Basel, Basel, Switzerland; Intensive Care Medicine, University Hospital Basel, Basel, Switzerland; Medical Faculty of the University of Basel, Basel, Switzerland; Department of Clinical Research, University of Basel, Basel, Switzerland; Department of Cardiac Surgery, University Hospital Basel, Basel, Switzerland; Medical Faculty of the University of Basel, Basel, Switzerland

**Keywords:** Infective endocarditis, *Staphylococcus aureus*, Cardiac surgery, Haemoadsorption, Sepsis

Dear Editor,

With great interest, we have read the recently published retrospective study by Haidari *et al.* where the authors found a strong positive effect of intraoperative haemadsorption on clinical outcomes in cases of confirmed *Staphylococcus aureus* endocarditis undergoing cardiopulmonary-bypass surgery [1]. However, we have concerns regarding the validity of the results:

1. Selection of the control group and specification of the cohort
  It needs to be clarified how the patients and controls for intraoperative haemoadsoprtion were selected. An implicit or explicit non-random allocation of haemoadsorption introduces a major selection bias and renders the results of the presented study challenging to interpret. Also, it should be clarified, how the present cohort differs from the data published in *PLoS One* in 2022 [2]. The publication of the study protocol might further clarify the study design.
2. Exclusion of tricuspid valve infectious endocarditis patients
  The authors excluded patients with tricuspid valve endocarditis, which is more often associated with intravenous drug use, intracardiac devices and central venous catheters and might thus be an important selection bias due to the exclusion of a predominantly multimorbid population [3]. This limitation should be adequately discussed in the limitations.
3. Large effect size
  The effect size seems very large, e.g. the 90-day mortality was reduced by 18.7% by the use of haemoadsorption, which results in a number needed to treat (NNT) of 5.3 patients to prevent 1 patient from dying [1]. As a comparison, the introduction of penicillin reduced the mean mortality rate of penicillin-sensitive diseases (e.g., pneumonia, diphtheria and scarlet fever) by 28% resulting in a NNT of 3.6 [4].
  In addition, the recently published REMOVE randomized controlled trial did not find any survival benefit (secondary outcome) when intraoperatively using the same haemoadsorption device in infectious endocarditis patients undergoing cardiac surgery [5]. Our study group also published retrospective data without an association of intraoperative haemoadsorption and any positive outcome in infective endocarditis [6]. Therefore, we suggest revising the existing discussion by providing a more inclusive view of available literature.
4. The wording of the ‘Take Home Message’
  In the ‘Take Home Message’ of the visual abstract, the authors conclude, ‘Intraoperative haemoadsorption improves clinical outcomes in patients after surgery for active Staphylococcus aureus endocarditis’. However, there is a large consensus in the medical research community that retrospective studies can only show association and are insufficient to prove causation. Hence, we recommend a more modest wording focusing on association.
5. Role of the manufacturer
  One of the authors (Prof. D. Wendt) is an employee of Cytosorbents Inc. (Berlin, Germany), which manufactures the assessed haemoadsorption device. However, the authors omitted to specify the role of the manufacturer in the design, conduction, and analysis of the present paper, which is a major limitation that should be discussed. Furthermore, it should be disclosed that Dr. Zaki Haidari, i.e. the first author of the present study, has been a speaker at the CytoSorb^®^ World Users Meeting organized by Cytosorbents in Berlin on 2 July 2022 (https://world-cytosorb-users-meeting.b2match.io/page-4331, accessed 26 February 2023).

**Conflict of interest:** Dr. Simon A. Amacher received a research grant from the Mach-Gaensslen Foundation Switzerland outside the present work. Mr. Jules Miazza received a research grant from the Fondation Andreas Paul Naef. Prof. Martin Siegemund has no conflict of interest in relation to the present letter. Until 2018, Dr. David Santer was a site-leader for patient inclusion into the international Cytosorb-Registry (www.cytosorb-registry.org) at the Department of Cardiovascular Surgery, Hospital Hietzing, Vienna, Austria, which was supported by CytoSorbents Europe GmbH (paid to the Karl Landsteiner Institute for Cardiac and Vascular Research, Vienna, Austria). Outside the present work, Dr. David Santer has received speaker honoraria and educational grants from Abbott and Medtronic, speaker honoraria from Abiomed, Nycomed GmbH and Zimmer GmbH and research grants from Medtronic (Schweiz) AG, Mussler Medical Supply (MMS) SA, Microport CRM, ‘Freiwillige Akademische Gesellschaft’, Basel and Fondation Andreas Paul Naef.
